# MetALD: New Perspectives on an Old Overlooked Disease

**DOI:** 10.1111/liv.70017

**Published:** 2025-04-03

**Authors:** Gustavo Ayares, Luis Antonio Diaz, Francisco Idalsoaga, Naim Alkhouri, Mazen Noureddin, Ramon Bataller, Rohit Loomba, Juan Pablo Arab, Marco Arrese

**Affiliations:** ^1^ Departamento de Gastroenterología Escuela de Medicina, Pontificia Universidad Católica de Chile Santiago Chile; ^2^ Escuela de Medicina, Universidad Finis Terrae Santiago Chile; ^3^ MASLD Research Center, Division of Gastroenterology and Hepatology University of California San Diego California USA; ^4^ Division of Gastroenterology Department of Medicine Schulich School of Medicine, Western University & London Health Sciences Centre London Ontario Canada; ^5^ Department of Hepatology Arizona Liver Health Chandler Arizona USA; ^6^ Houston Methodist Hospital Houston Texas USA; ^7^ Liver Unit Hospital Clinic and Institut d'Investigacions Biomediques August Pi I Sunyer (IDIBAPS) Barcelona Spain; ^8^ Division of Gastroenterology, Hepatology, and Nutrition, Department of Internal Medicine Virginia Commonwealth University School of Medicine Virginia USA

**Keywords:** alcoholic liver disease, alcohol‐related liver disease, ametabolic dysfunction‐associated steatotic liver disease, cirrhosisalcoholic cirrhosis, MASLD, NAFLD, non‐alcoholic fatty liver disease

## Abstract

Metabolic dysfunction‐associated steatotic liver disease (MASLD) and alcohol‐associated liver disease (ALD) are the major contributors to the liver disease burden globally. The rise in these conditions is linked to obesity, type 2 diabetes, metabolic syndrome and increased alcohol consumption. MASLD and ALD share risk factors, pathophysiology and histological features but differ in their thresholds for alcohol use, and the ALD definition does not require the presence of metabolic dysfunction. A recent multi‐society consensus overhauled the nomenclature of liver steatosis and introduced the term MetALD to describe patients with metabolic dysfunction who drink more than those with MASLD and less than those with ALD. This new terminology aims to enhance the understanding and management of liver disease but poses challenges, such as the need to accurately measure alcohol consumption in research and clinical practice settings. Recent studies show that MetALD has significant implications for patient management, as it is associated with increased mortality risks and more severe liver outcomes compared to MASLD alone. MetALD patients face increased risks of liver disease progression, cancer and cardiovascular disease. The diagnosis of MetALD involves the adequate quantification of alcohol use through standardised questionnaires and/or biomarkers as well as proper assessment of liver disease stage and progression risk using non‐invasive tools including serologic markers, imaging, elastography techniques and genetic testing. Effective management requires addressing both metabolic and alcohol‐related factors to improve outcomes. This review intends to provide a comprehensive overview of MetALD, covering pathogenesis, potential diagnostic approaches, management strategies and emerging therapies.

**Summary**

- A recent multi‐society consensus overhauled the nomenclature of liver steatosis and introduced the term MetALD to describe patients with metabolic dysfunction who drink beyond the thresholds set for Metabolic dysfunction‐associated steatotic liver disease (MASLD) but less than patients with alcohol‐associated liver disease (ALD).
- Diagnosis of MetALD poses several challenges due to the arbitrary nature of thresholds for alcohol use, which require further validation in prospective studies; the known impact of alcohol consumption on cardiometabolic risk factors; and the need for a comprehensive history of alcohol use that considers recent and lifetime alcohol intake using available tools (i.e., questionnaires and biomarkers).
- Prevalence of MetALD in the general population ranges from 2.1% to 8.3% depending on the setting studied. Within patients with steatotic liver disease (SLD), the estimated overall prevalence of MetALD is 10%. Preliminary data suggest that patients with MetALD have an increased risk of all‐cause, cancer and liver‐related mortality compared with those without SLD.
- The management of MetALD necessitates a comprehensive approach that addresses the coexistence of cardiometabolic risk factors and alcohol use. Drugs proven to be effective in MASLD, such as resmetirom and glucagon‐like peptide 1 (GLP‐1) receptor agonists, should be tested in patients with MetALD.

**Abbreviations**
AASLD: American Association for the Study of Liver Diseases
AAT: alpha‐1 antitrypsin
AGA: American Gastroenterology Association
AIH: autoimmune hepatitis
ALD: alcohol‐associated liver disease
ALT: alanine aminotransferase
AMA: anti‐mitochondrial antibodies
ANA: antinuclear antibodies
ASMA: anti‐smooth muscle antibodies
AST: aspartate aminotransferase
AUD: alcohol use disorder
AUDIT: Alcohol Use Identification Test
AUDIT‐C: Alcohol Use Identification Test Consumption
BAC: blood alcohol concentration
BMI: body mass index
CAP: controlled attenuation parameter
CDT: carbohydrate deficient transferrin
ChREBP: carbohydrate response element binding protein
CIDEB: cell death‐inducing DNA fragmentation factor‐like effector b
DNL: de novo lipogenesis
DSM‐5: Diagnostic and Statistical Manual of Mental Disorders fifth edition
EMA: anti‐endomysial antibodies
EtG: ethyl glucuronide
EtP: ethyl phosphate
EtS: ethyl sulfate
FAEE: fatty‐acid ethyl esters
FAT: fatty acid translocase
FATP2‐5: fatty acid transport proteins 2 and 5
FDA: Food and Drug Administration
FFA: free fatty acid
FIB‐4: fibrosis‐4
FMT: faecal microbiota transplantation
GLP‐1: glucagon‐like peptide 1
HBV: hepatitis B virus
HCC: hepatocellular carcinoma
HCV: hepatitis C virus
LKM‐1: liver kidney microsome type 1
LSM: liver stiffness measurement
LT: liver Transplantation
MASH: metabolic dysfunction‐associated steatohepatitis
MASLD: metabolic dysfunction‐associated steatotic liver disease
MBOAT7: membrane bound O‐acyltransferase domain containing 7
MetALD: metabolic dysfunction and alcohol‐related liver disease
MRE: magnetic resonance elastography
MRI: magnetic resonance imaging
NAFLD: non‐alcoholic fatty liver disease
NCD: non‐communicable disease
NHANES: National Health and Nutrition Examination Survey
NIAAA: National Institute on Alcohol Abuse and Alcoholism
PDFF: proton density fat fraction
PEth: phosphatidylethanol
PNPLA3: patatin like phospholipase domain 3
SLD: steatotic liver disease.
SREBP‐1c: sterol regulatory element‐binding protein 1c
T2DM: type 2 diabetes mellitus
TM6SF2: transmembrane 6 superfamily member 2
tTG‐IgA: tissue transglutaminase antibodies
UK: United Kingdom
UNOS: United Network for Organ Sharing
US: United States
VLDL: low‐density lipoprotein
WHO: World Health Organization

## 1 Introduction

Metabolic dysfunction‐associated steatotic liver disease (MASLD) and alcohol‐associated liver disease (ALD) are the leading causes of the burden of liver disease globally [1, 2]. Both conditions also represent the most frequent causes of steatotic liver disease (SLD) in contrast to other etiologies, such as hepatitis C virus (HCV) chronic infection, Wilson's disease and rare monogenic diseases [3]. The spread of the Western diet, unhealthy habits and food insecurity have led to an epidemic of obesity and type 2 diabetes mellitus (T2DM) [4], while levels of alcohol consumption are persistently elevated in most countries [5, 6, 7, 8]. These epidemiological trends have contributed to a significant increase in SLD prevalence [5, 9, 10], as well as changes in the burden of cirrhosis, hepatocellular carcinoma (HCC) and related conditions [11, 12, 13, 14, 15]. Although other relevant etiologies of liver disease, such as HCV infection, have been globally addressed and strategies are on course to eliminate them in the upcoming decades, SLD is still under‐appreciated by governmental organisations and most physicians [16, 17]. Unfortunately, there is suboptimal awareness of liver steatosis as a relevant health problem, leading to neglecting associated risks and to lack of proper patient's assessment [17]. Importantly, prior evidence has demonstrated that even isolated steatosis is independently associated with significantly increased overall mortality and this risk increased progressively with worsening MASLD histology [18, 19, 20].

**FIGURE 1:**
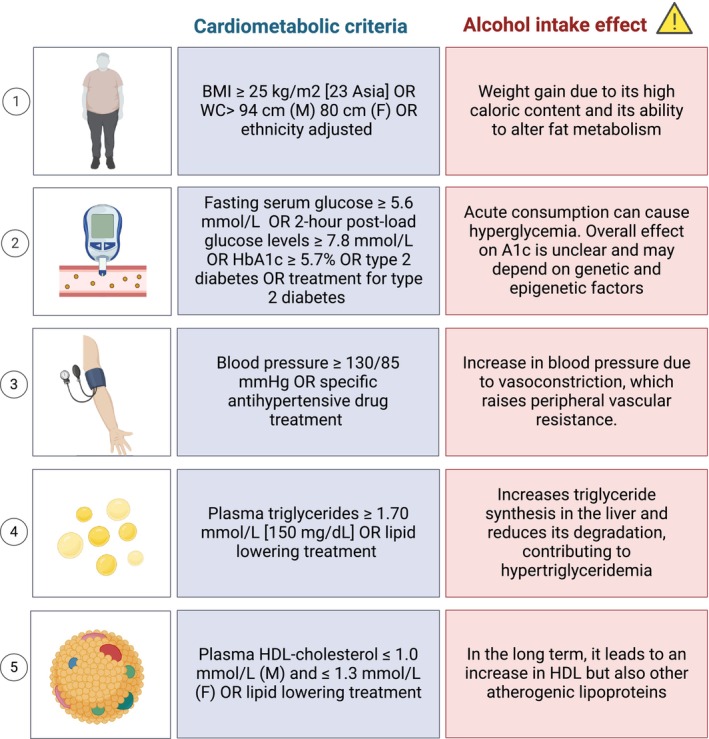
Impact of Alcohol on cardiometabolic criteria used for MASLD diagnosis. In patients with hepatic steatosis, the presence of at least one of the five factors is required to make the diagnosis of MASLD. When applying these criteria for diagnosis, the effects of alcohol on blood pressure, hypertriglyceridemia and hyperglycaemia should be taken into account. BMI, body mass index; F, female; HbA1C, glycated haemoglobin; M, male; WC, waist circumference [24].

Since its first description in 1980, MASLD [previously known as non‐alcoholic fatty liver disease (NAFLD)] has been considered a different entity than ALD [21]. However, they share several features, and the distinction between them is based on the amount of consumed alcohol, which has been arbitrarily established [22]. Of note, MASLD and ALD have overlapping pathophysiology, share genetic and epigenetic factors, have a similar spectrum of key histological features (spanning from isolated steatosis to steatohepatitis and cirrhosis), and have similar clinical features, with a proportion of patients lying at the intersection of both conditions [23, 24]. In 2023, the multi‐society consensus statement on SLD nomenclature established objective criteria for metabolic dysfunction, allowing the diagnosis of MASLD (Figure 1) [25]. This nomenclature acknowledged that, in some individuals, alcohol may act as a synergistic driver of liver injury, proposing the term MetALD [25]. This acronym was proposed to describe individuals with metabolic dysfunction and alcohol intake beyond the thresholds allowed for MASLD, but that do not meet the criteria set for ALD diagnosis. Thus, MetALD is used to categorise those subjects that met MASLD criteria and report alcohol intake between 140 and 350 g/week in women and 210–420 g/week in men [25]. Although these diagnostic criteria, and in particular the thresholds for alcohol use, require further validation in prospective studies, the concept of MetALD has been regarded as an opportunity to understand the role of alcohol in SLD development and progression, as well as to gauge patients at risk of more severe outcomes [26]. However, MetALD also presents challenges, such as the appropriate consideration of how alcohol consumption impacts cardiometabolic risk factors (Figure 1), such as high blood pressure, hypertriglyceridemia and hyperglycaemia, which are integral to the MASLD definition [24]. Furthermore, challenges arise from the accuracy and consistency of alcohol consumption data collected in previous MASLD studies, particularly regarding the quality of assessments for both current and lifetime alcohol consumption [24, 27]. Moreover, individual alcohol consumption rates fluctuate throughout life. Current definitions of MetALD rely on static evaluations, which may not accurately reflect real‐world clinical practice. Finally, the literature has not yet adequately addressed the implications of reclassifying patients based on changes in their current alcohol consumption. Although important advances have been made in our understanding of the combined effects of metabolic factors and alcohol intake on the onset and progression of chronic liver disease, the effect on the natural history of the disease needs to be better characterised [28, 29]. To date, studies of the interactions between alcohol consumption and different components of metabolic syndrome have shown that the coexistence of alcohol intake and obesity or metabolic syndrome contributes to the development of liver disease at least in an additive fashion [23, 30]. Also, patients with obesity and heavy alcohol use have higher levels of steatosis, inflammation, fibrosis or cirrhosis compared to their lean and abstinent counterparts [31]. In this review, we discuss key aspects related to MetALD and its impact on the development and progression of chronic liver disease. Current views on clinical assessment and potential therapeutic strategies for this new liver disease category are also reviewed in light of recent data.

## 2 Pathogenesis of ALD and MASLD: Differences and Similarities

As previously stated, MASLD and ALD share multiple pathophysiological mechanisms that activate hepatic inflammatory and fibrogenic pathways, leading to hepatocellular injury, fibrosis progression and liver function derangement [23]. A central key mechanism is the disruption of lipid handling by hepatocytes, characterised by an imbalance between lipid uptake, synthesis, degradation and export of free fatty acids (FFAs) [32, 33]. This leads to the formation of large lipid microvesicles (i.e., lipid droplets) comprising a neutral lipid core (primarily containing triacylglycerol and sterol esters) surrounded by a phospholipid monolayer and proteins [34]. In the case of MASLD, hepatic lipid accumulation is a consequence of a systemic condition, the major metabolic perturbations of which are increased adiposity and insulin resistance [35]. These conditions lead to augmented FFA influx to the liver and upregulation of FFA transporters [e.g., fatty acid translocase protein CD36 and fatty acid transport proteins 2 (FATP2) and 5 (FATP5)], resulting in fatty acid accrual [15, 36]. Notably, insulin resistance and the consumption of high loads of fructose, which is common in people with MASLD, directly drive hepatic de novo lipogenesis (DNL), a crucial regulator of intrahepatic triglyceride content and a critical factor in hepatic steatosis development [37, 38]. It has been estimated that DNL accounts for up to 38% of the fatty acids stored as triglycerides in MASLD [39]. In the case of ALD, although alcohol can influence adipose tissue lipolysis, it is primarily considered a direct liver toxin that disrupts multiple facets of liver homeostasis [40, 41]. Regarding lipid metabolism, chronic alcohol consumption directly increases FFA uptake and hepatic DNL in hepatocytes, while simultaneously decreasing mitochondrial β‐oxidation and very low‐density lipoprotein (VLDL) secretion, thus promoting net increased hepatic lipid accumulation [32, 41]. While increased uptake of circulating FFA is related to increased expression and function of the fatty acid translocase (FAT/CD36), DNL is induced by the activation of a myriad of transcription factors, such as sterol regulatory element‐binding protein 1c (SREBP‐1c), early growth response‐1 and carbohydrate response element binding protein (ChREBP) [42, 43]. VLDL secretion is impaired due to altered lipoprotein assembly, and decreased mitochondrial β‐oxidation may result from several mechanisms, including altered NADH:NAD+ ratio, inhibition of carnitine palmitoyltransferase 1 activity and carnitine deficiency [32, 44].

The relationship between insulin sensitivity and alcohol is complex. Experimental evidence suggests that low doses may have a net positive effect on insulin resistance in women, whereas binge drinking and chronic alcohol consumption are associated with impaired insulin signaling [45, 46]. Human studies on the impact of alcohol on insulin sensitivity and glycaemia suggest that alcohol may decrease fasting insulin and glycated haemoglobin (HbA1c) concentrations among nondiabetic subjects, but its overall effects remain unclear [47]. Importantly, these effects may be influenced by individual factors, including genetic background and gender [48].

Once steatosis is established, liver injury can occur in both MASLD and ALD through a myriad of mechanisms, including mitochondrial dysfunction, endoplasmic reticulum stress, generation of toxic metabolites such as ceramides (leading to cell death) and the activation of immune responses, resulting in inflammation, steatohepatitis and liver disease progression [23, 40, 49]. Multiple other pathways can promote liver damage in both MASLD and ALD, and certainly in MetALD. These include disturbances of the gut‐liver axis and altered gut microbiome composition, dysregulation of bile acid metabolism, hepatic sinusoidal endothelial cell dysfunction, hepatic stellate cell activation and dysregulation of homeostatic mechanisms of repair in the liver [23, 34, 50]. Mitochondrial dysfunction, in particular, seems to play an important role in the mutual interaction of MASLD and alcohol abuse (i.e., MetALD) [51]. Indeed, genetic and epigenetic regulation of the above‐mentioned events and other phenomena such as the activation of inflammatory signals and cell death pathways modulate the magnitude of liver injury and ultimately liver disease progression to fibrosis and cirrhosis [52, 53, 54]. A detailed description of these overlapping mechanisms of liver injury in MASLD and ALD is beyond the scope of this review. The reader is referred to recent reviews on the topic for further details [23, 34, 40].

## 3 New Fatty Liver Disease Nomenclature: Strengths and Controversies

In 2023, a multi‐society consensus was carried out to redefine the terminology regarding fatty liver disease [25]. SLD was chosen as an overarching term to encompass the various etiologies of steatosis [55]. MASLD was coined to encompass patients with hepatic steatosis and at least one of five cardiometabolic risk factors (Figure 1). Alcohol use allowed to diagnose MASLD was defined as lower than 140 and 210 g per week in women and men, respectively. The term metabolic dysfunction‐associated steatohepatitis (MASH) was coined to designate patients with MASLD and steatohepatitis, which includes a subset of patients at higher risk of advancing to more severe liver disease stages and adverse outcomes. ALD was defined by a weekly alcohol intake of over 350 and 420 g in women and men, respectively. As mentioned earlier, MetALD was defined by the presence of metabolic dysfunction and levels of alcohol use per week greater than 140 g/week and 210 g/week for women and men, respectively [25]. If weekly consumption is closer to 140/210 g/week in women and men, respectively, the predominant phenotype is metabolic dysfunction. If it is closer to 350/420 g/week, then MetALD is considered ALD predominant. However, clear thresholds have not been established, and it is considered that MetALD encompasses a spectrum between MASLD and ALD. This continuum was reflected in a recent study showing dose‐dependent interaction of alcohol with cardiometabolic risk factors in patients with MASLD and MetALD [29]. In this study, even 100 g/week up to 130 g/week in women and 200 g/week in men is associated with significant fibrosis in up to 25.5% of MASLD patients depending on the number of cardiometabolic criteria present in a given patient [29]. In another study, MetALD patients shared some characteristics with MASLD, but they showed more characteristics of ALD patients [56].

Although MASLD diagnostic criteria are distinct from the former NAFLD criteria, several studies report a nearly complete overlap between the MASLD‐defined population and the historical NAFLD‐defined populations, allowing the use of both terms indistinctly [57]. The change in nomenclature also emphasises that identification of patients having coexisting risk factors for SLD favours a more holistic approach and highlights the role of alcohol use as a contributor to the development and progression of SLD [24, 58]. Indeed, while the new nomenclature for fatty liver diseases offers potential advantages in terms of clarity and risk stratification, it also presents challenges that need to be addressed through ongoing research and clinical validation. Key areas of focus include: improving the accuracy of alcohol use quantification methods, conducting real‐world studies to assess the impact of alcohol on cardio metabolic criteria, studies assessing metabolic dysfunction and alcohol use over time, and better delineation of the use of non‐invasive diagnostic techniques in different SLD categories. This information will be crucial in refining this classification and improving patient outcomes.

## 4 Prevalence of SLS, MASLD and MetALD and Impact on Survival

Since the proposal of MetALD as a newly recognised category of SLD, some studies have assessed its prevalence in primary care settings and nationwide databases. For instance, MetALD prevalence has been estimated at 2.5% in the United States (US) [3]. Also, a Korean study including 2535 individuals who underwent magnetic resonance elastography (MRE) and magnetic resonance imaging (MRI) proton density fat fraction (PDFF) during health check‐ups in five primary care health promotion clinics estimated an SLD prevalence of 39.1% with MASLD prevalence of 29.5%, MetALD 7.8% and ALD 0.3% [59]. Another study using the United Kingdom (UK) biobank data of 40,189 patients estimated an SLD prevalence of 27% [60]. Of them, 89% had MASLD, 7.9% had MetALD, and only 1% had ALD. In this cohort, patients with MASH had the highest magnetic resonance imaging–estimated proton density fat fraction (MRI–PDFF) and the highest body mass index (BMI). MetALD had similar prevalence in men and women with obesity but was absent in women without obesity [60].

**FIGURE 2:**
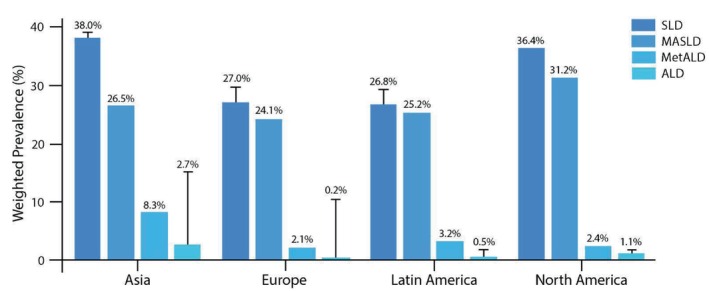
Weighted prevalence of SLD and subgroups in different regions. There are differences in the prevalence of SLD, MASLD, MetALD and ALD across different regions of the world according to recent reports. According to new classifications, causes of SLD may include viral, genetic and other metabolic causes accounting for the differences by region [3, 59, 60, 61, 62, 63, 64, 65, 66]. ALD, alcohol‐associated liver disease; MASLD, metabolic dysfunction‐associated steatotic liver disease; SLD, steatotic liver disease.

The weighted prevalence of SLD and subgroups in different regions is presented in Figure 2. There are differences in the prevalence of SLD, MASLD, MetALD and ALD across different regions of the world according to recent reports [3, 59, 60, 61, 62, 63, 64, 65, 66]. A recent systematic review and meta‐analysis assessing the prevalence, characteristics and outcomes of patients with MetALD showed that the pooled overall prevalence of this entity among the SLD population was 10% (95% CI: 7%–13%) [66].

With regard to prognosis, a population‐based cohort study in the US showed that patients with MetALD showed increased all‐cause, cancer and liver‐related mortality risk compared with those without SLD [67]. This trend was more pronounced in the MetALD individuals with advanced fibrosis estimated by the Fibrosis‐4 (FIB‐4) Index [67]. Other recent studies suggest that MetALD is associated with an increased risk of mortality compared to MASLD alone [68, 69, 70]. In a systematic review, higher levels of alcohol use increased the risk of malignancy and cancer‐related mortality in patients with MetALD over those with MASLD [71]. Another study from Korea also showed that MASLD and MetALD are associated with an increased risk of cancer, particularly liver and gastrointestinal cancers [72]. Furthermore, in a more recent systematic review, patients with MetALD were found to be at increased risk of all‐cause [HR 1.44 (95% CI: 1.24–1.66)], cardiovascular disease [HR 1.17 (95% CI: 1.12–1.21)] and cancer‐related mortality [HR 2.07 (95% CI: 1.32–3.25)] compared to non‐SLD individuals [66]. Finally, a retrospective cohort study using the United Network for Organ Sharing (UNOS) [73] registry estimated that MetALD is the third leading aetiology among those waitlisted and transplanted, exhibiting worse pre‐ and post‐transplantation outcomes compared to ALD [73]. Patients with MetALD also experienced higher waitlist removal, all‐cause mortality and graft failure than those with ALD [73]. Thus, while MASLD‐related mortality seems to be driven by the fibrosis stage and associated cardiovascular risk [74], alcohol use, even at lower levels, could increase the risk of all adverse outcomes including several malignancies [11, 75].

## 5 Recognition and Assessment of MetALD

**FIGURE 3:**
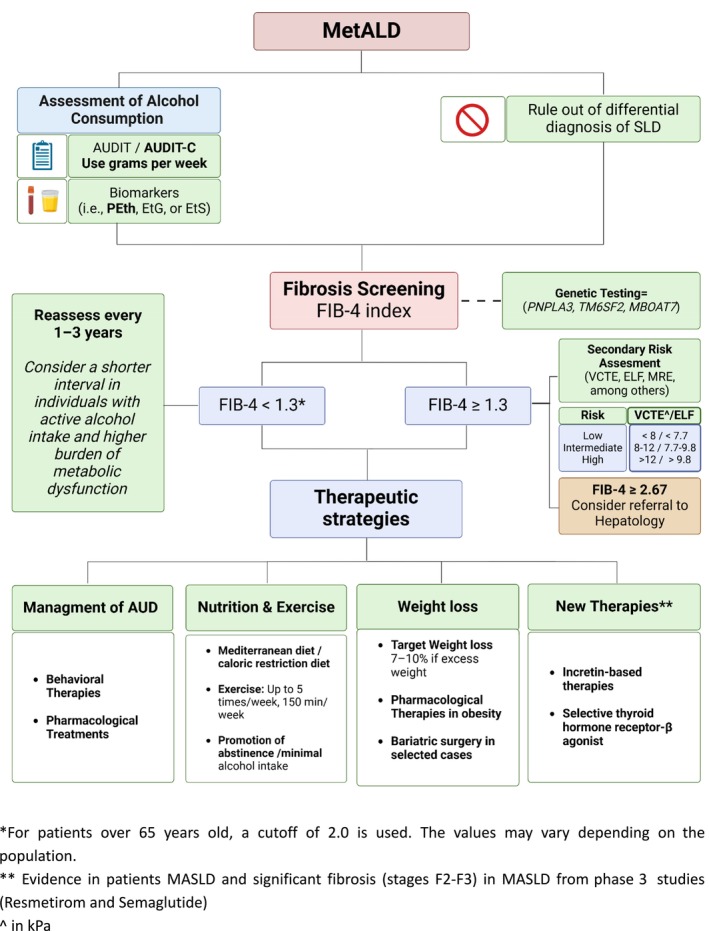
Systematic approach to patients with MetALD. In patients with MetALD, it is initially important to obtain a comprehensive history of alcohol use consumption. The use of validated questionnaires (AUDIT‐C) and biomarkers can aid in the detection and precise quantification of alcohol use. Ruling out other causes of steatosis is also an important diagnosis step. Subsequently, screening for fibrosis using the Fibrosis‐4 (FIB‐4) index is in order, followed by a second test or follow‐up based on the results. Treatment should then be initiated, including managing AUD and ALD, nutrition and exercise prescription, promotion of weight loss and starting specific therapies when necessary. ALD, alcohol‐related liver disease; AUD, alcohol use disorder; ELF, enhanced liver fibrosis; EtG, ethyl glucuronide; EtS, ethyl sulfate; PEth, phosphatidylethanol; VCTE, vibration‐controlled transient elastography.

Due to the high prevalence of SLD in the general population and the heterogeneity of its spectrum, a personalised approach to manage patients with suspected SLD should be attempted, considering the dominant drivers of disease in patient subgroups [76]. The initial assessment to identify the aetiology and individuals at risk of more advanced disease can be challenging. Firstly, all patients with SLD should be assessed for metabolic dysfunction, interrogated meticulously for alcohol use and evaluated for other causes of liver disease according to recent guidelines [24, 57, 77] In the following paragraphs, we summarise the most relevant steps in the SLD assessment in outpatient clinical practice (Figure 3) with emphasis on MetALD recognition.

### 5.1 Assessment of Steatosis, Steatohepatitis and Other Causes of SLD

Steatosis can be determined by using liver ultrasound (US), computed tomography (CT), controlled attenuation parameter (CAP), MRI‐PDFF and liver biopsy [78]. Liver US is one of the most accepted screening methods for steatosis because it is non‐invasive, inexpensive and widely available [79]. However, it is operator‐dependent and cannot differentiate fibrosis from steatosis accurately [80]. Its sensitivity and specificity to identify steatosis range between 60%–94% and 88%–95%, respectively [81]. Controlled attenuation parameter (CAP) is a non‐invasive tool to measure ultrasound attenuation when travelling through steatotic tissue and is incorporated into the Fibroscan (Echosens, Paris, France) equipment, but it is operator‐dependent and the cost of equipment is higher than that of the conventional US [82]. In ALD, CAP can identify mild, moderate and severe steatosis with an area under the receiver operating characteristic curve of 0.77, 0.78 and 0.82, respectively [83]. In addition, a CAP ≥ 290 dB/m ruled in any steatosis with 88% specificity and 92% positive predictive value. To date, studies that validate sensitivity and sensibility of CAP in MetALD have not been performed. Finally, MRI‐PDFF is a non‐invasive method with an adequate correlation with liver steatosis estimated by liver biopsy [84], widely available, reproducible and could guide treatment since a ≥ 30% relative decline in MRI‐PDFF is associated with higher odds of histologic response and MASH resolution [85].

While steatohepatitis is a significant driver of MASLD progression, current guidelines do not consider its presence, independent of fibrosis stage, as a factor in treatment decisions [77]. Assessment of steatohepatitis is generally performed using liver biopsy, which in the setting of SLD is indicated in the context of clinical trials or diagnostic uncertainty. In the majority of cases, liver biopsy is not required for clinical management of individuals with MASLD, which is also applicable for MetALD and ALD [77, 86].

Detection of liver steatosis must trigger a thorough investigation into the specific cause(s) of liver fat accumulation in each individual patient [78]. A careful evaluation of cardiometabolic risk factors, associated conditions and comorbidities as well as a precise assessment of alcohol intake (see below) is in order. Besides metabolic dysfunction and alcohol consumption, consideration should be given to drug‐induced liver steatosis and other less frequent liver diseases. These include chronic hepatitis C virus infection, hemochromatosis, Wilson disease and other conditions such as celiac disease and autoimmune liver disorders that can coexist with hepatic steatosis. Thus, proper assessment of these conditions is indicated [78].

### 5.2 Assessing Alcohol Use and Drinking Pattern

Patients with suspected SLD must be thoroughly evaluated for active and/or former alcohol use [24]. There are several methods to identify patients at risk of ALD or alcohol use disorder (AUD), including standardised questionnaires, direct and indirect biomarkers of alcohol use (see below) Table 1. All of these methods have benefits and limitations and have been validated in different populations. Although quantification of alcohol use levels may seem easy in clinical practice, there are several gaps to overcome. Standard drinks are not universally accepted as some guidelines recommend using 10 grams [87] of alcohol per drink while others suggest using 14 g [88, 89]. Thus, a standard drink may vary from 8 to 20 g. The Dietary Guidelines for Americans consider that 14 g of alcohol is roughly the same as 350 mL (12 fl oz) of beer (5% weight/volume), 150 mL (5 fl oz) of wine (12%–13% weight/volume) or 45–50 mL of liquor (1.5 fl oz) (40%–45% weight/volume) [90]. Notably, clinicians often do not routinely ask for the number and type of drinks, nor the drinking pattern in clinical practice [91].

### 5.3 Screening Questionnaires

There are several questionnaires based on self‐reports to screen the presence of AUD (Table 1). One option is a single‐question screener: ‘How many times in the past year have you had 5 or more drinks in a day (for men) or 4 or more drinks in a day (for women)?’ [99]. A second option is the concise three questions questionnaire AUDIT‐C (Alcohol Use Disorders Identification Test Consumption). These screening questions should be followed up with more extensive questionnaires to identify AUD and a careful quantification of alcohol consumption by day or week and pattern of consumption [100, 101, 102]. The AUDIT questionnaire was developed by the World Health Organization (WHO) and is appropriate for patients with liver disease [92]. This instrument comprises 10 questions with a specific scoring system, and the first three questions are related to quantity‐frequency measures, providing retrospective estimates of average or usual consumption. An AUDIT score higher than 8 is considered a positive screening for AUD [92], while a cutoff of 15 for men and 13 for women have a 100% specificity but low sensitivity (20% and 18%, respectively) for detecting alcohol dependence [103]. AUDIT‐C of over 3 in men and 4 in women has a 73% sensitivity and 91% specificity in women and 86% sensitivity and 89% specificity in men to predict alcohol misuse [93]. Unfortunately, underreporting of alcohol consumption is common and will always be a clinical challenge due to stigmatisation and social perception of alcohol consumption. Therefore, all questions in this area must be without stigma or prejudice to offer a comfortable space in which to address the issue. For example, up to 29% of patients diagnosed with presumed MASLD had positive hair biomarkers for alcohol use [96]. The AUDIT‐C demonstrated significantly lower performance in detecting alcohol use compared to alcohol biomarkers, emphasising the need for broader biomarker utilisation in routine clinical practice, which can be useful to reveal the presence/amount of recent alcohol intake. Of note, certain vulnerable populations, such as younger individuals and those with non‐routine drinking patterns, may be more likely to underreport alcohol consumption [104].

**TABLE 1:** Tools for assessing alcohol consumption in clinical practice.

Methods	Tools	Characteristics
Screening questionnaires	Alcohol Use Identification Test (AUDIT) [92]	10‐question survey developed by the World Health Organization (WHO) that evaluates alcohol consumption, related symptoms and the consequences of drinking. A total score of 8 or more suggests the presence of alcohol‐related problems
	Alcohol Use Identification Test Consumption (AUDIT‐C) [93]	A shortened version of the AUDIT focusing on the first three questions related to alcohol consumption. Useful as an initial screening tool in clinical settings to identify risky drinking patterns Risky alcohol consumption is indicated by a score of 4 or more in men and a score of 3 or more in women
	CAGE Questionnaire [94]	Brief 4‐question survey used to identify potential problems with alcohol consumption. It is simple to administer and effective for screening for alcohol use disorders A score of 2 or more out of 4 may indicate a potential problem with alcohol use and suggests that further evaluation is warranted
Biomarkers	Phosphatidylethanol (PEth) [95]	A phospholipid, formed exclusively in the presence of ethanol and found in blood, is considered a highly sensitive and specific biomarker for chronic alcohol consumption with a long detection window (up to 2–4 weeks)
	Ethyl Glucuronide (EtG) [95, 96]	A direct metabolite of ethanol, detectable in urine for 24 to 72 h after alcohol consumption, is commonly used to detect recent alcohol use
	Ethyl Sulfate (EtS) [97]	Metabolite of ethanol found in urine, used alongside EtG for detecting alcohol use. Can provide additional information about recent drinking behaviour
	Carbohydrate Deficient Transferrin (CDT) [95]	CDT is a form of transthyretin with a reduced carbohydrate content resulting from chronic alcohol consumption. Elevated CDT levels are used to detect heavy alcohol use or recent alcohol consumption within a detection window of 14–17 days
	Fatty Acid Ethyl Esters (FAEE) [95]	FAEE can be detected in biological samples like blood, urine and hair for days to weeks after alcohol consumption, making them useful for monitoring chronic drinking patterns
	Mean Corpuscular Volume (MCV) [95]	Chronic alcohol consumption can lead to macrocytic anaemia, which is reflected by an increased MCV. This can be a marker of alcohol‐related damage to bone marrow or nutrient deficiencies associated with chronic drinking
	Gamma‐Glutamyl Transpeptidase (GGT) [95]	GGT activity is elevated in individuals with alcohol use and is often used in conjunction with other tests to diagnose alcohol‐related liver disease. However, GGT levels can also be elevated due to various factors, including biliary obstruction, diabetes and certain medications
	AST/ALT ratio [98]	An increased AST/ALT ratio can be particularly indicative of alcohol‐related liver disease, although it may also suggest advanced liver disease in the absence of heavy drinking

### 5.4 Biomarkers of Alcohol Use

While not yet validated for diagnosing MetALD, non‐oxidative products of alcohol metabolism can be detected, including ethyl glucuronide, ethyl sulphate, ethyl phosphate, phosphatidylethanol (PEth) and fatty‐acid ethyl esters (Table 1) [95, 97]. Other indirect biomarkers of alcohol use include carbohydrate deficient transferrin (CDT), aspartate aminotransferase (AST)/alanine aminotransferase (ALT) ratio > 2, elevated mean corpuscular volume and gamma‐glutamyl transpeptidase [105]. These tools should complement traditional alcohol history assessments, including interviews and collateral information. Among all these biomarkers, PEth has the most promising profile to identify individuals at risk of MetALD [24, 105]. In particular, PEth is not significantly influenced by sex or body mass index, and levels ≥ 72 ng/mL correlate with an intake of 26 g of pure ethanol with a 90% sensitivity and 66% specificity [106]. Further studies have shown that a PEth threshold of approximately 200 ng/mL may detect consumption up to 60 g/day, aligning with the upper threshold for MetALD [24]. While reliable, PEth's clinical applicability may be limited by cost and accessibility constraints. Furthermore, additional research is needed to establish optimal thresholds for identifying patients with MetALD in clinical settings [24].

## 6 Risk Stratification in MetALD

Recent guidelines provide a clear set of recommendations to diagnose, stratify and manage patients with MASLD and ALD [57, 77, 86]. Since the degree of liver fibrosis has been shown to be the main driver of liver disease progression and a predictor of adverse outcomes in both MASLD and ALD, non‐invasive assessment of advanced fibrosis is a key step in the risk stratification of patients [77, 86]. Importantly, some data suggest that patients with MetALD may have a significantly higher degree of liver fibrosis and hence a potentially worse prognosis than MASLD patients [29, 59]. Of note, a recent study conducted in the United States with prospective enrolled community‐dwelling overweight and obesity participants showed that those with MetALD have a prevalence of advanced fibrosis of up to 7.7% and cirrhosis at 3.9% [107]. Thus, liver fibrosis is frequently observed in MetALD, and its detection is relevant as it could lead to treatment and decision‐making.

In the field of MASLD, in addition to liver fibrosis assessment, consideration should be given to other factors such as age (i.e., > 50 years) the number of cardiometabolic risk factors, additional comorbidities (e.g., obstructive sleep apnoea and polycystic ovary syndrome), ethnicity and genetic factors as they may determine increased risk of disease progression [77]. These concepts fully pertain to patients with MetALD. Additional remarks on fibrosis screening and genetic testing are provided below.

### 6.1 Fibrosis Screening

Liver fibrosis can be assessed with a variety of tools including serum biomarkers, either patented [e.g., enhanced liver fibrosis test (ELF)] and non‐patented tests (e.g., FIB‐4), estimation of liver stiffness measurement (LSM) using vibration‐controlled transient elastography (VCTE) and image‐based techniques such as ultrasound‐based elastography or MRE [98, 108]. Although MRE has the highest diagnostic accuracy in detecting each stage of fibrosis, it is less available in clinical practice [109]. Importantly, VCTE and MRE have demonstrated an impact in predicting relevant clinical outcomes over time in patients with MASLD in some studies [110, 111]. Although evidence supporting the use of these techniques in MASLD is abundant, albeit of moderate quality, data is limited in the setting of ALD and scarce in the case of MetALD [108].

According to guidelines, individuals with metabolic risk factors or signs of SLD, including those with MetALD, should be initially stratified using FIB‐4, and those with high FIB‐4 values should be referred to a second test to further screen for significant liver fibrosis [i.e., assessment of liver stiffness with VCTE or evaluation of fibrosis with the ELF test] [57, 77]. Patients with FIB‐4 > 2.67 or altered results in the second test should be referred to specialised care (Figure 3). In this scenario, the role of the gastroenterologist or hepatologist should be focused on the exclusion of other liver diseases, determining the need for liver biopsy, administration of specific treatment for comorbidities (i.e., chronic viral hepatitis, autoimmune liver diseases, among others), fibrosis management and follow‐up, and finally management of patients cirrhosis and referral to liver transplant. Some guidelines also include patients with persistently elevated aminotransferases (> 6 months) in the group of patients that should be followed up by a gastroenterologist or hepatologist. Close communication with other healthcare providers, such as endocrinologists, general practitioners, nurse practitioners and nutritionists, facilitates multidisciplinary management of metabolic comorbidities including interventions that may also improve liver injury [112].

The American Association for the Study of Liver Diseases (AASLD) algorithm for the evaluation of patients with SLD suggests that in settings with a low prevalence of advanced fibrosis, such as in the primary care setting, the emphasis is on excluding advanced fibrosis using a test with a high negative predictive value. When the FIB‐4 is < 1.3, patients can be followed in the primary care setting and reassessed periodically. Patients without 1 or more metabolic risk factors can be reassessed every 2–3 years. It is important to emphasise, as noted above, that while evidence supporting the use of FIB‐4 in MASLD is ample, data for its use in ALD is more limited [113]. According to the ACG Clinical Guideline on Alcohol‐Associated Liver Disease, FIB‐4 represent a useful initial non‐invasive test for assessing fibrosis in individuals with ALD, although its utility may be reduced due to factors such as predominant AST elevation and thrombocytopenia caused by active alcohol use [86]. For MetALD, evidence is emerging. A recent study demonstrated that the FIB‐4 score performs well as a screening tool for advanced hepatic fibrosis in MetALD, showing similar diagnostic accuracy to that in MASLD without significant alcohol intake [114]. Patients with MASLD or MetALD should receive frequent FIB‐4 monitoring. These tests can be performed at the time of diagnosis and repeated at intervals of 6 months to 2 years, depending on fibrosis stage and the patient's response to intervention. In patients older than age 65, a FIB‐4 cutoff of > 2.0 should be used and patients under 35 should undergo secondary assessment as FIB‐4 has low accuracy in those patients [112]. Importantly, liver elastography may have reduced accuracy in obese patients (BMI > 35). MRE should be considered as a more robust assessment tool in this population [115]. The use of MRI is more accurate in measuring hepatic fat content and is not affected by patient factors, aetiology of liver disease, and concomitant liver abnormalities (i.e., iron overload, liver inflammation) [116]. However, it has a higher cost and longer examination time than other techniques. The sensitivity and specificity of MRI‐PDFF to detect liver steatosis ≥ 5% are 86% and 83%, respectively [117]. Patients with confirmed cirrhosis should undergo guideline‐approved follow‐up that includes screening for HCC, clinically significant portal hypertension and state of compensation. The specifics of this management are beyond the scope of this review and can be found elsewhere [118, 119].

### 6.2 Genetic Testing

The rates of fibrosis progression and hepatic decompensation in both MASLD and ALD vary depending on baseline disease severity, genetic background, individual environmental and comorbid disease determinants [26]. The effects of alcohol might also differ according to the presence of obesity, which increases the risk of cirrhosis in a dose‐dependent manner [23, 120]. The role of genetic polymorphisms influencing the natural course of both diseases is a field of growing interest in the context of personalised precision medicine [121, 122]. The patatin like phospholipase domain 3 (*PNPLA3*) I148M genetic variant is the most extensively studied genetic factor associated with an increased prevalence and severity of MASLD [123]. Polymorphisms in other genes encoding proteins involved in hepatocyte lipid metabolism have also been associated with influencing the clinical course of MASLD [54]. Of note, variants (e.g., E167K), in both the transmembrane 6 superfamily member 2 (*TM6SF2*) gene, that regulate the assembly and secretion of VLDL, and polymorphisms of the membrane‐bound O‐acyltransferase domain containing 7 (*MBOAT7*) gene, which influences phospholipid metabolism, have also been associated with an increased risk of MASLD and its progression to more severe forms, including fibrosis and steatohepatitis [54]. Other gene variants seem to be protective, such as hydroxysteroid 17‐beta dehydrogenase 13 (*HSD17B13*) [124], a gene that encodes an enzyme that also localises to lipid droplets in hepatocytes and cell death‐inducing DNA fragmentation factor‐like effector b (*CIDEB*) [125], a protein needed for the activation of de novo lipogenesis. These single nucleotide polymorphisms have been identified as significant factors influencing the outcomes of MASLD and, in some cases (e.g., *PNPLA3* and *HSD17B13 genes*), of ALD and the risk of increased liver fibrosis and progression to end‐stage liver disease [54]. A longitudinal study assessing the impact of genetic polymorphisms on outcomes in patients with T2DM and MASLD who were positive for *PNPLA3* and *TM6SF2* risk alleles (combined polymorphism analysis) demonstrated an even higher risk of cirrhosis if two or more risk alleles were present (odds ratio [OR] 18.48). Regarding cirrhosis complications, polymorphisms individually and additively impacted SLD severity, with an increased risk of cirrhosis and its complications (OR 27.20) [126]. Also, individuals with ALD who are homozygous for the *PNPLA3* I148M genetic variant have a 2.2–2.4‐fold risk of cirrhosis [127, 128]. Additionally, the *ALDH2* rs671 polymorphism has been linked to poor prognosis in ALD‐related hepatocellular carcinoma [129].

Although integrating genetic testing into clinical risk prediction seems like a logical step in risk stratification in MASLD and ALD, until now there is not a clear demonstration that genetic information plays an independent and additional role in clinical risk prediction. Thus, current societal guidelines do not yet recommend routine screening due to lack of robust data indicating that genetic diagnosis has an impact on decision‐making [112]. Studies assessing the role of genetic factors specifically in patients with MetALD are anticipated.

## 7 Therapeutic Strategies in MetALD

The management of MetALD necessitates a comprehensive approach that addresses the coexistence of cardiometabolic risk factors and alcohol use, underscoring the importance of tailored treatment strategies to improve patient outcomes [24, 27]. Patients with MetALD would benefit from a comprehensive model of care involving a multidisciplinary approach that integrates prevention, diagnosis, treatment and long‐term management of cardiometabolic conditions with proper evaluation and treatment of AUD, if present [23, 130].

Not all treatment recommendations proposed in recent MASLD guidelines are necessarily applicable to the MetALD population, and specific data is needed in this regard, particularly regarding pharmacological therapy. All patients should undergo nutritional assessment and a plan established for regular follow‐up [27, 131]. The need for more specialised obesity management, including glucagon‐like peptide 1 (GLP‐1) receptor agonists and other incretin therapies, bariatric surgery referral, behavioural therapy, and additional cardiology or lipid metabolic support, should be assessed on an individual basis and local availability [132, 133, 134]. The same stands for the management of AUD, which may require the participation of addiction specialists [131]. In the following paragraphs, we discuss the most relevant and promising therapies for MetALD.

## 8 Nutrition and Exercise

Achieving and sustaining weight loss, as part of lifestyle changes to improve peripheral insulin sensitivity, is challenging. Fewer than 10% of patients achieve effective weight loss despite structured interventions at 1 year, and less than half of these maintain the weight lost 5 years after intervention and require ongoing multidisciplinary care [135]. This should include support systems, family engagement and behavioural medicine specialists. Current AASLD guidelines recommend changes in dietary composition (e.g., low‐carbohydrate vs. low‐fat diets, saturated vs. unsaturated fat diets, intermittent fasting, Mediterranean diet, etc.) and different intensities of caloric restriction to improve SLD [112]. Coffee consumption, independent of caffeine content, may also be beneficial. Drinking 3 or more cups per day could be recommended in the absence of contraindications based on the reduced risk for SLD and liver fibrosis demonstrated in epidemiological studies and meta‐analyses with a low quality of evidence [136]. Chronic alcohol misuse can lead to malnutrition due to inadequate intake of macronutrients (primary malnutrition) and eventually malabsorption of dietary intake (secondary malnutrition) although the effect of moderate alcohol use on nutrition is not clear. In patients with advanced liver disease, regular snacking (each 2–3 h) and night snacking are encouraged to prevent protein malnourishment.

Patients with MetALD should be strongly encouraged to increase their activity level to the extent possible independent of weight loss, as this has hepatic and cardiometabolic benefits (at least five times per week for a total of 150 min/week) [112]. In particular, those individuals with predominant metabolic dysfunction can benefit from regular exercise, a decrease in liver fat content and cardiovascular risk factors [137, 138], while those with higher levels of alcohol use and AUD could benefit from stopping the progression of liver disease and a decrease in markers of liver apoptosis [139, 140].

### 8.1 Bariatric Surgery

Bariatric surgery can resolve steatohepatitis, improve fibrosis and induce sustained weight loss of up to 30% with an impact on all‐cause morbidity and mortality [141]. Although currently accepted criteria for bariatric surgery are BMI ≥ 35 kg/m^2^ irrespective of metabolic comorbid disease or BMI between 30 and 35 kg/m^2^ and an obesity‐related condition (i.e., T2DM or pre‐DM, uncontrolled hypertension, osteoarthritis of hip or knee, urinary incontinence) [142]. MASLD is increasingly accepted as a comorbid condition benefitting from bariatric surgery [143]. There is no specific evidence for MetALD; however, extrapolating data from MASLD is reasonable, although abstinence should be considered mandatory. Bariatric surgery should be considered as a therapeutic option in patients who meet criteria for metabolic weight loss surgery [112]. However, the efficacy of bariatric surgery in patients with well‐compensated MetALD cirrhosis is not established. An important consideration about bariatric surgery in patients with MetALD is that it has been associated with a higher risk of AUD and ALD [144]. Moreover, a recent study shows that people undergoing bariatric surgery and binge‐drinking are more prone to suicide and liver‐related mortality [145]. Thus, a proper psychiatric evaluation and follow‐up before and after the procedure is essential to increase the successful rate of this procedure but also to prevent the development of AUD [146, 147].

## 9 Promising Therapies in MetALD

Although many therapies are being developed for MASLD and for ALD, as well as for cirrhosis with varying degrees of success, in this review we shall highlight three main treatments of note that could apply to MetALD. In particular, although some of these therapies have been approved by the Food and Drug Administration (FDA) to treat individuals with T2DM or obesity, they are not approved to treat MASLD as the leading indication of therapy [112, 148].

Incretin‐based therapies have an important effect on the landscape of obesity treatment [149, 150]. In addition, GLP‐1 receptor agonists have also demonstrated a potential effect in decreasing levels of alcohol use, being a potential therapeutic strategy in patients with MetALD [151]. A phase 2 trial (LEAN study) using liraglutide (GLP‐1 receptor agonist) 1.8 mg daily compared to placebo showed a reduction in MASH activity without worsening of liver fibrosis [152]. In addition, in a phase 2 randomised controlled trial using subcutaneous semaglutide (GLP‐1 receptor agonist) at a dose of 0.1, 0.2 or 0.4 mg daily or placebo, patients on semaglutide had a dose‐dependent effect on MASH, with resolution achieved with no worsening of fibrosis in up to 59% over placebo [132]. Additionally, recent results from a phase 3 trial of semaglutide (Essence trial) in patients with MASH‐related fibrosis showed evidence of efficacy in improving liver histology [153, 154]. Interestingly, a recent report showed that GLP‐1 RA use was associated with a lower risk of progression to cirrhosis and mortality among patients with MASLD and diabetes [155]. Other dual or triple agonists, including tirzepatide [156], survodutide [133], pemvidutide [157] and retratutride [158] have shown promising results in MASH resolution and reduction in body weight, being attractive therapeutic agents for MetALD. Also, a current randomised trial is assessing the combination of semaglutide and cagrilintide (a dual amylin and calcitonin receptor agonist) in adults with excess weight, AUD and liver disease (ClinicalTrials.gov ID: NCT06409130).

Resmetirom is an oral liver‐directed, thyroid hormone receptor beta‐selective agonist developed for the treatment of steatohepatitis with significant fibrosis. Both the 80‐mg dose and the 100‐mg dose of resmetirom were superior to placebo (29% vs. 9.7%) with respect to MASH resolution and improvement in liver fibrosis by at least one stage. Furthermore, low‐density lipoprotein (LDL) levels were improved (16.3% vs. 0.1%) over placebo [159]. Although this study was conducted using the previous definitions of NAFLD, a post hoc analysis identifying potential MetALD patients using CDT > 2.5% and/or a PEth > 20 ng/mL had a similar response to resmetirom to those without evidence of alcohol use [160].

Both human and experimental studies have clearly shown that the gut‐brain axis plays a key role in modulating human behaviour [161, 162]. In those with chronic liver disease, regardless of the presence of metabolic dysfunction or excessive alcohol use, the gut‐liver axis is further modulated by impaired liver function [163, 164]. Therefore, targeting gut microbiota composition and function offers an interesting alternative for the development of MetALD therapies [151, 165]. For example, a phase 1 placebo‐controlled trial showed that one faecal microbiota transplantation (FMT) enema was safe in patients with cirrhosis over 6 months and reduced alcohol craving and consumption using objective biomarkers short‐term (15 days post‐FMT) with higher short‐chain fatty acids and beneficial stool microbiota composition [166].

## 10 Special Consideration: MetALD in Lean Individuals

As mentioned, MASLD is observed predominantly in people with obesity or other metabolic dysfunctions; however, 7%–20% of individuals with MASLD have a BMI under 25 kg/m^2^ [167]. The American Gastroenterology Association (AGA) published a guidance on appropriate clinical evaluation in lean individuals with MASLD suggesting that these patients should be viewed as at high risk for inherited or genetic disorders, lipodystrophy, drug‐induced SLD and inflammatory disorders [167]. However, these were published before the change in nomenclature and therefore must be recontextualised to reflect the current understanding of MetALD. In lean subjects with steatosis, the identification of underreported alcohol intake over the threshold levels for MetALD is essential to ensure a proper management of AUD. Physicians must query about alcohol use patterns, including the number of drinking days per week, the maximum number of drinks consumed in 24 h and binge‐drinking behaviour. Importantly, some studies have shown that the *PNPLA3* risk variant is more common among individuals with lean MASLD than their overweight and obese counterparts [168]. However, more data is needed in this regard. Additionally, other causes of ‘lean MASLD’ can be related to high fructose or high‐fat diets, visceral obesity, changes in body composition (e.g., lipodystrophy in HIV and non‐HIV persons), celiac disease or rare congenital abnormalities such as lysosomal acid lipase deficiency (LAL‐D), familial hypobetalipoproteinaemia B and abetalipoproteinaemia [169]. Drugs should always be evaluated as a cause of SLD in lean individuals. Thus, considerations should be given to the use of methotrexate, amiodarone, tamoxifen and corticosteroids, among others. Other conditions to consider are hepatotropic viruses (HCV genotype 3), nutritional and gastrointestinal tract‐related factors (celiac disease, total parenteral nutrition), endocrine disorders (hypothyroidism) and toxin exposures (vinyl chloride) [167]. Lean individuals with SLD should be risk‐stratified and screened for fibrosis or cirrhosis. The AGA also recommends that a liver biopsy should be considered if there is uncertainty regarding contributing causes of liver injury and/or the stage of liver fibrosis.

Regarding outcomes, while lean individuals with MASLD may have a better metabolic profile, they often face a higher risk of mortality and severe liver disease compared to their overweight and obese counterparts [169]. The role of alcohol in modulating this risk needs to be addressed.

## 11 Research Gaps and Future Perspectives

The introduction of MetALD into the spectrum of SLD represents a significant step towards a better understanding of the pathophysiology, clinical features and treatment of individuals exhibiting overlapping features of MASLD and ALD. Future research efforts should address several key areas that remain inadequately understood. Firstly, more granular data on the natural history of the MetALD patient subgroup is necessary to elucidate how moderate and harmful alcohol consumption influences disease progression and outcomes. Secondly, further data are required to ascertain the dose‐dependent impact of alcohol on cardiovascular and malignancy risk in patients with MetALD [170]. Furthermore, precise definitions of optimal alcohol use assessment methodologies are crucial, and further research is imperative to establish a ‘safe’, if any exists, threshold for alcohol consumption. This necessitates more granular data on the effects of varying alcohol consumption levels and drinking patterns in patients with MASLD and MetALD. All this data is needed to effectively inform policy decisions and for the design of clinical trials. Additionally, further research is needed on how to effectively integrate genetic assessments, such as the detection of *PNPLA3* genotypes or other relevant genetic variants, into clinical risk prediction models [57, 159, 171]. Finally, the refinement of preclinical models, incorporating alcohol in conjunction with metabolic stressors, would contribute to a more accurate replication of the complex pathophysiology observed in the setting of MetALD [172].

In 2013 the 66th World Health Assembly, member states adopted a resolution setting an ambitious global target of a 25% relative reduction in premature mortality from non‐communicable diseases (NCDs) along the cardiometabolic and respiratory spectrums by 2025, mainly considering NCD risk factors (i.e., physical inactivity, unhealthy diet, tobacco and alcohol) and four disease areas (i.e., cardiovascular disease, diabetes, chronic respiratory disease and cancer). However, in this plan, SLD was not considered [173]. Moreover, most countries do not have a national policy or action plan addressing SLD, particularly MASLD [88, 174, 175, 176]. Policies targeting obesity, as well as those addressing alcohol use could impact the burden of MetALD in the long term and should be encouraged [174, 177, 178, 179, 180]. If implemented, public policies to reduce the harmful use of alcohol would also impact the MetALD burden [181]. Thus, societal efforts are needed to put MASLD/MetALD and ALD within key public health priorities.

In conclusion, to deliver precision medicine, we must be prepared to address all underlying conditions and proactively prevent disease progression. MetALD exemplifies this challenge, representing a condition that combines two of the leading causes of liver disease, MASLD and ALD. While both MASLD and ALD are relatively well‐characterised conditions with robust evidence to guide specific and actionable clinical recommendations, it is still less clear how the combination of the two influences each other. Whether MetALD is a subgroup with a higher risk of adverse outcomes is likely, but yet to be confirmed. Also, how we address dynamic changes in the patient's lifetime and our understanding of the disease trajectory is somewhat limited. Nevertheless, compelling evidence supports the notion that the combination of metabolic dysfunction and moderate alcohol consumption, two prevalent real‐world conditions, may present a unique and significant clinical challenge.

## Author Contributions

**Gustavo Ayares:** investigation, writing – original draft, writing – review and editing, creation of figures/tables. **Luis Antonio Diaz:** investigation, writing – original draft, writing – review and editing, creation of figures/tables. **Francisco Idalsoaga:** conceptualization, writing – review, editing, creation of figures and tables. **Naim Alkhouri:** conceptualization, writing – review, editing. **Mazen Noureddin:** conceptualization, writing – review, editing. **Ramon Bataller:** conceptualization, writing – review, editing. **Rohit Loomba:** conceptualization, writing – review, editing. **Marco Arrese:** conceptualization, writing – review, editing and supervision. **Juan Pablo Arab:** conceptualization, writing – review, editing and supervision.

## Conflicts of Interest

R.B. consults for Glaxo, Smith and Klein, Novo Nordisk and Boerimhem Inhelheim. R.L. serves as a consultant to Aardvark Therapeutics, Altimmune, Anylam/Regeneron, Amgen, Arrowhead Pharmaceuticals, AstraZeneca, Bristol‐Myer Squibb, CohBar, Eli Lilly, Galmed, Gilead, Glympse bio, Hightide, Inipharma, Intercept, Inventiva, Ionis, Janssen Inc., Madrigal, Metacrine Inc., NGM Biopharmaceuticals, Novartis, Novo Nordisk, Merck, Pfizer, Sagimet, Theratechnologies, 89 bio, Terns Pharmaceuticals and Viking Therapeutics. In addition, his institutions received research grants from Arrowhead Pharmaceuticals, AstraZeneca, Boehringer‐Ingelheim, Bristol‐Myers Squibb, Eli Lilly, Galectin Therapeutics, Galmed Pharmaceuticals, Gilead, Intercept, Hanmi, Intercept, Inventiva, Ionis, Janssen, Madrigal Pharmaceuticals, Merck, NGM Biopharmaceuticals, Novo Nordisk, Merck, Pfizer, Sonic Incytes and Terns Pharmaceuticals. He is a co‐founder of LipoNexus Inc. M.A. consults for Inventiva and Astrazeneca and has served as a speaker for Siemens.

## Data Availability

Data sharing is not applicable to this article, as no new data were created or analyzed in this study.
